# Pulmonary restriction predicts long-term pulmonary impairment in people with HIV and tuberculosis

**DOI:** 10.1186/s12890-020-01368-4

**Published:** 2021-01-07

**Authors:** Sara C. Auld, Hardy Kornfeld, Pholo Maenetje, Mandla Mlotshwa, William Chase, Mboyo di-Tamba Vangu, Drew A. Torigian, Robert S. Wallis, Gavin Churchyard, Gregory P. Bisson

**Affiliations:** 1 Departments of Medicine and Epidemiology, School of Medicine and Rollins School of Public Health, Emory University, Atlanta, GA USA; 2 Department of Medicine, University of Massachusetts Medical School, Worcester, USA; 3 The Aurum Institute, Johannesburg, South Africa; 4 Perelman School of Medicine at the University of Pennsylvania, Philadelphia, PA USA; 5 Department of Nuclear Medicine, CM Johannesburg Academic Hospital, University of the Witwatersrand, Johannesburg, South Africa; 6 School of Public Health, University of Witwatersrand, Johannesburg, South Africa; 7 Department of Biostatistics, Epidemiology, and Informatics, Center for Clinical Epidemiology and Biostatistics, Perelman School of Medicine at the University of Pennsylvania, Philadelphia, PA USA

**Keywords:** Tuberculosis, HIV, Respiratory function tests, Pulmonary disease

## Abstract

**Background:**

While tuberculosis is considered a risk factor for chronic obstructive pulmonary disease, a restrictive pattern of pulmonary impairment may actually be more common among tuberculosis survivors. We aimed to determine the nature of pulmonary impairment before and after treatment among people with HIV and tuberculosis and identify risk factors for long-term impairment.

**Methods:**

In this prospective cohort study conducted in South Africa, we enrolled adults newly diagnosed with HIV and tuberculosis who were initiating antiretroviral therapy and tuberculosis treatment. We measured lung function and symptoms at baseline, 6, and 12 months. We compared participants with and without pulmonary impairment and constructed logistic regression models to identify characteristics associated with pulmonary impairment.

**Results:**

Among 134 participants with a median CD4 count of 110 cells/μl, 112 (83%) completed baseline spirometry at which time 32 (29%) had restriction, 13 (12%) had obstruction, and 9 (7%) had a mixed pattern. Lung function was dynamic over time and 30 (33%) participants had impaired lung function at 12 months. Baseline restriction was associated with greater symptoms and with long-term pulmonary impairment (adjusted odds ratio 5.44, 95% confidence interval 1.16–25.45), while baseline obstruction was not (adjusted odds ratio 1.95, 95% confidence interval 0.28–13.78).

**Conclusions:**

In this cohort of people with HIV and tuberculosis, restriction was the most common, symptomatic, and persistent pattern of pulmonary impairment. These data can help to raise awareness among clinicians about the heterogeneity of post-tuberculosis pulmonary impairment, and highlight the need for further research into mediators of lung injury in this vulnerable population.

## Background

Among the approximately 8.7 million people who survive tuberculosis disease each year, it is estimated that 30–50%, representing 2.6–4.4 million individuals, will develop chronic lung disease [1–6]. Reduced lung function has been associated with increased all-cause mortality and lost productivity and so the potential impact for millions of tuberculosis survivors is profound [7–9]. In fact, the majority of tuberculosis-related disability is attributable to chronic pulmonary impairment after cure and not acute disability during active disease [1]. While there is growing awareness of the long-term burden of pulmonary impairment after tuberculosis, there are limited data from prospective cohorts to help us understand the nature of these respiratory sequelae.

Post-tuberculosis lung impairment has traditionally been viewed as an obstructive phenomenon, similar to chronic obstructive pulmonary disease (COPD), where patients have difficulty exhaling as a result of inflammation and damage to the large airways. However, recent data suggest that pulmonary restriction may be as common, if not more so, among tuberculosis survivors [10–12]. Pulmonary restriction, which is characterized by the inability to inhale to full lung capacity, typically arises from scarring and fibrosis of the lung parenchyma. Treatment for pulmonary obstruction focuses on relieving airflow limitation with bronchodilators and inhaled corticosteroids. In contrast, there are limited treatments available for fibrotic pulmonary diseases, none of which have been studied in patients with tuberculosis [13].

People with HIV are a uniquely vulnerable population in their potential for lung injury. Not only are people with HIV at increased risk of developing tuberculosis, they also have an increased risk for non-infectious pulmonary diseases such as COPD and pulmonary fibrosis [14–16]. Furthermore, while people with HIV and pulmonary tuberculosis often have less lung involvement at diagnosis, immune restoration on antiretroviral therapy (ART) may lead to incident pulmonary inflammation and declines in lung function [17, 18]. In a limited number of studies that have focused on lung impairment after tuberculosis in people with HIV, it is unclear whether HIV coinfection alters the risk of incident pulmonary impairment [2, 12, 19, 20].

Given the disproportionate burden of tuberculosis in people with HIV, there is an urgent need to better understand the epidemiology, patterns, and drivers of lung damage in people with HIV and tuberculosis. We sought to determine the prevalence of obstructive versus restrictive lung impairment at baseline and after tuberculosis treatment in a cohort of individuals with HIV and tuberculosis coinfection in order to identify characteristics associated with persistently impaired lung function. A better understanding of pulmonary impairment among tuberculosis survivors will lay the groundwork for future trials to improve lung function after tuberculosis.

## Methods

### Study design and participants

The Lung Function after TB-IRIS (LIFT-IRIS) Study was a prospective cohort study conducted in Gauteng, South Africa that evaluated pulmonary function and symptoms in HIV-infected adults diagnosed and treated for pulmonary tuberculosis [18]. Results are reported here in accordance with STROBE guidelines. We enrolled adults age 18 years or older who were newly diagnosed with HIV and ART-naïve with a CD4 count ≤ 500 cells/μL. Pulmonary tuberculosis was diagnosed based upon a positive Xpert MTB/RIF® test. Exclusion criteria included rifampin-resistance, tuberculous meningitis, current immunosuppressive therapy, current incarceration, and pregnancy. All participants were treated with standard first-line tuberculosis treatment and ART (i.e., efavirenz, tenofovir, emtricitabine) as per local guidelines. Participants were clinically screened for additional opportunistic infections at all study visits.

### Study procedures

Study visits occurred prior to ART initiation (baseline) and at 6 and 12 months. At baseline, participants were asked about smoking and cigarette pack-years (i.e., cigarettes smoked/day multiplied by years smoked) were calculated. CD4 cell count and HIV viral load were measured at baseline and 1 month; hemoglobin A1c (HbA1c) was measured at baseline. At each visit, pulmonary symptoms were assessed using the COPD Assessment Test (https://www.catestonline.org) [21, 22], a 6 min walk test was conducted [23], and spirometry was performed using the EasyOne Pro® Spirometer (nDD Medical Technologies, Andover, MA, USA). See supplemental methods for further details of the spirometry testing.

In accordance with the Global Initiative for Chronic Obstructive Lung Disease (GOLD) guidelines, we classified participants as having airflow obstruction on the basis of a forced expiratory volume in 1 s (FEV_1_)/forced vital capacity (FVC) ratio less than 70% [22]. Restriction was defined as an FVC less than 80% of predicted, and participants were classified as having a mixed pattern of both obstruction and restriction if the FEV_1_/FVC ratio and the FVC were below 70% and 80% predicted, respectively.

^18^F-fluorodeoxyglucose (FDG)-positron emission tomography/computed tomography (PET/CT) scans were done at baseline on the first 50 participants who agreed to participate in a radiology sub-study. Data regarding the PET findings are reported elsewhere [18]. For this analysis, the CT images from these scans were reviewed for evidence of lung damage, including bronchiectasis, bronchiolitis, and cavitation, using a score for mycobacterial infections developed by Song et al. with possible CT scores ranging from zero (i.e., no abnormal findings) to a maximum of 44. See supplemental methods and Additional file 1: Table S2 for further details of the PET/CT scans and their scoring [24].

### Statistical analysis

We determined the relationship between clinical covariates and pulmonary impairment using bivariate analyses and logistic regression. Categorical and continuous variables were compared using a chi-square and Kruskal–Wallis test, respectively, and Pearson coefficients were calculated for correlations between continuous variables. Cohen’s kappa coefficient was used to determine agreement between spirometry diagnoses at different time points. Logistic regression models were created to estimate (1) the association between participant characteristics and impaired lung function at baseline and (2) the association between participant characteristics at baseline (including baseline lung function) and impaired lung function at 12 months. All variables included in the models were baseline variables, with the exception of the lung function data and data on the CD4 cell count and viral load (which were characterized both at baseline and as the change from baseline to 4 weeks). Variables were included in the multivariate models on the basis of purposeful selection of clinical covariates, bivariate association, and directed acyclic graphs [25]. Analyses were conducted in SAS version 9.4 (SAS Institute, Cary, NC) and R version 3.6.0.

### Ethics

All participants provided written informed consent. The Institutional Review Boards of the University of Pennsylvania, the University of Witwatersrand, and the Health Research Ethics Counsel in South Africa approved this study.

## Results

There were 134 participants enrolled in the study with a median age of 36 (interquartile range [IQR] 31–43), 57 (43%) of whom were female, and all of whom reported Black African race (Table 1). While 54 (40%) participants reported ever smoking, with a median of 2.8 pack-years smoked (IQR 1.2–7.5), only 21 (16%) reported current smoking at enrollment. No participants reported chronic lung disease, and two participants reported mine work of 1 year. At baseline, the median CD4 count was 110 cells/μL (IQR 49–194) and the median HIV viral load was 146,742 copies/mL (IQR 54,194–494,851).

**Table 1:** Sociodemographic and clinical characteristics of study participants at baseline (n = 134)

Characteristic	n (%) or median (IQR)
Age	36 (31–43)
Female	57 (43)
Black African race	134 (100)
BMI	19.1 (17.9–21.8)
Current smoker	21 (16)
Ever smoker	53 (40)
Pack-years of cigarettes	2.8 (1.2–7.5)
Chronic lung disease	0 (0)
History of mine work	2 (1)
HbA1c	6.1 (5.8–6.4)
CD4 cell count (cells/μl)	110 (49–194)
HIV viral load (copies/ml)	146,742 (54,194–494,851)
FEV_1_% predicted	79 (66–89)
FVC % predicted	85 (73–95)
FEV_1_/FVC ratio	79 (73–85)
Time from TB treatment to ART initiation (days)	24 (15–42)

*ART* antiretroviral therapy, *BMI* body mass index, *FEV*_*1*_ forced expiratory volume in 1 s, *FVC* forced vital capacity, *HbA1c* hemoglobin A1c, *IQR* interquartile range

112 (83%) participants completed spirometry testing at baseline. Approximately half of those (58, 52%) had normal lung function, while the remaining half met criteria for pulmonary impairment: 13 (12%) had obstruction, 32 (29%) had restriction, and 9 (7%) had a mixed pattern.

### Pulmonary restriction has greater symptoms and radiologic involvement at baseline

**Fig. 1:**
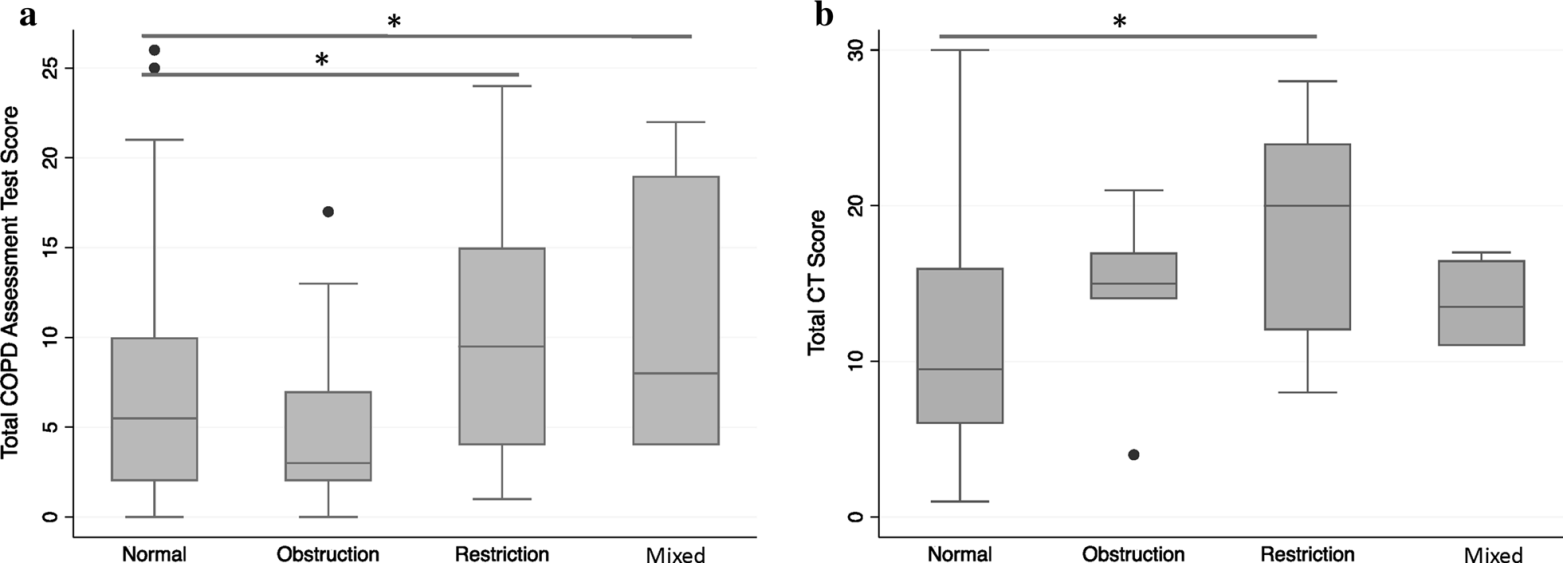
**a** Baseline COPD assessment test (CAT) scores and **b** baseline CT radiologic scores according to baseline diagnosis (*p value < 0.05)

At baseline, pulmonary symptoms were moderately inversely correlated with both FEV_1_ (ρ = -0.28, *p* value 0.004) and FVC (ρ = − 0.28, *p* value 0.003), such that participants with worse symptoms tended to have a lower FEV_1_ and FVC (Additional file 1: Figure S1). Participants with either restriction or a mixed pattern had a significantly greater symptom burden than those with normal spirometry (median [IQR] COPD Assessment Test score 10 [4–15] for restriction and 8 [2, 4–18] for mixed vs. 4 [2–9] for normal), including greater cough, chest tightness, breathlessness, and lower energy levels. In contrast, participants with obstruction did not differ with respect to symptom burden when compared to those with normal spirometry (Fig. 1a and Table 2). The median 6 min walk test distance was also 35 m shorter for participants with restriction than those with normal spirometry, although this difference was not statistically significant (*p* value 0.32; minimum clinically important difference = 26 m) [26].

**Table 2:** Pulmonary symptoms on the COPD Assessment Test (CAT) and 6MWT distance at baseline according to the pattern of baseline pulmonary function

	Normal (n = 58)	Obstruction (n = 13)	Restriction (n = 32)	Mixed (n = 9)
Total CAT score	4 (2–9)	3 (2–7)	10 (4–15)*	8 (4–19)*
Cough	1 (0–2)	1 (0–1)	2 (1–3)*	1 (1–2)
Phlegm	1 (0–1)	1 (0–1)	1 (0–2)	1 (1–2)
Chest tightness	0 (0–1)	0 (0–1)	0 (0–2)*	1 (0–2)*
Breathlessness	0 (0–2)	0 (0–1)	2 (0–3)*	1 (1–2)
Limited activity	0 (0–1)	0 (0–1)	0 (0–3)	1 (0–3)
Confidence leaving home	0 (0–0)	0 (0–0)	0 (0–0)	0 (0–1)
Sleep	0 (0–1)	0 (0–0)	0 (0–2)	0 (0–3)
Energy	1 (0–2)	1 (0–1)	2 (1–2)*	1 (0–2)
CAT 0 n (%)	6 (10)	1 (8)	0 (0)*	0 (0)
CAT 1–5	27 (47)	7 (54)	12 (38)	4 (44)
CAT 6–10	15 (26)	2 (15)	6 (19)	2 (22)
CAT > 10	10 (17)	3 (23)	14 (44)	3 (33)
6MWT distance	399 (360–447)	378 (307–456)	364 (326–442)	389 (332–422)

*CAT* COPD Assessment Test, score range 0–40, *6MWT* 6 min walk test

All categories of impaired lung function (i.e., obstruction, restriction, and mixed) were compared to normal and an asterisk (*) indicates a *p* value < 0.05 as compared to normal. All values are represented as median and interquartile range unless otherwise indicated

For the subset of 38 participants with both spirometry and a CT scan at baseline, the total CT score was also moderately inversely correlated with FEV_1_ (ρ = − 0.44, *p* value 0.006) and FVC (ρ = − 0.41, *p* value 0.01), such that those with greater radiologic involvement tended to have lower FEV_1_ and FVC (Additional file 1: Figure S2). As with symptoms, the total CT score for participants with obstruction did not significantly differ from those with normal spirometry, whereas those with restriction had significantly higher CT scores (median [IQR] for normal 10 [7–12] vs. obstruction 15 [14–17], *p* value 0.14 vs. restriction 20 [2, 12–23], *p* value 0.005) (Table 3 and Fig. 1b). When stratified according to the type of radiologic abnormality, those with obstruction had a significant increase in bronchiectasis as compared to those with normal spirometry (median [IQR] 8 [5–8] for obstruction vs. 2 [1–3] for normal). Those with restriction not only had more bronchiectasis (median [IQR] 5 [2–9] for restriction) but also had more bronchiolitis, consolidation, mosaic perfusion, lobar volume loss, and pleural effusions than those with normal spirometry.

**Table 3:** Radiologic abnormalities on CT scan according to the pattern of baseline pulmonary function

	Normal (n = 16)	Obstruction (n = 5)	Restriction (n = 13)	Mixed (n = 4)
Total CT score	10 (7–12)	15 (14–17)	20 (12–24)*	14 (11–17)
Bronchiectasis	2 (1–3)	8 (5–8)*	5 (2–9)*	4 (4–5)
Bronchiolitis	4 (2–5)	5 (5–6)	6 (5–6)*	5 (4–6)
Cavities	0 (0–2)	0 (0–0)	2 (0–3)	0 (0–0)
Nodules	1 (1–1)	1 (0–1)	1 (0–1)	1 (0–1)
Consolidation	1 (0–1)	1 (0–1)	2 (1–3)*	2 (1–2)
Bullae	0 (0–1)	0 (0–1)	0 (0–0)	0 (0–0)
Emphysema	0 (0–0)	0 (0–0)	0 (0–0)	0 (0–1)
Mosaic perfusion	1 (1–2)	1 (0–2)	2 (2–2)*	2 (1–2)
Lobar decrease	0 (0–0)	0 (0–0)	1 (0–1)*	1 (0–2)
Pleural effusion	0 (0–0)	0 (0–0)	0 (0–1)*	0 (0–1)

*CT* computed tomography, CT score range = 0–44

All categories of impaired lung function (i.e., obstruction, restriction, and mixed) were compared to normal and an asterisk (*) indicates a *p* value < 0.05 as compared to normal. All values are represented as median and interquartile range unless otherwise indicated

We next used logistic regression to estimate the association between pulmonary symptoms and impaired lung function at baseline. In bivariate analysis, having a higher baseline COPD Assessment Test score was significantly associated with restriction (odds ratio [OR] for 5-point increase in COPD Assessment Test score 1.77, 95% confidence interval [CI] 1.18–2.68) but not obstruction (OR 0.99, 95% CI 0.53–1.84) (Table 4). After adjusting for age, gender, baseline CD4 count and smoking, higher COPD Assessment Test scores continued to be associated with restriction but not obstruction (adjusted OR [aOR] for restriction 1.79, 95% CI 1.15–2.80; aOR for obstruction 0.88, 95% CI 0.45–1.72).

**Table 4:** Unadjusted and adjusted odds ratios for the association between baseline pulmonary symptoms as measured by the COPD Assessment Test (CAT) score and pulmonary obstruction or restriction as compared to normal lung function at baseline

	Baseline obstruction				Baseline Restriction			
	Unadjusted OR	95% CI	Adjusted OR^a^	95% CI	Unadjusted OR	95% CI	Adjusted OR^a^	95% CI
CAT score	0.99	0.53–1.84	0.88	0.45–1.72	**1.77**	**1.18–2.68**	**1.79**	**1.15–2.80**
Age	1.62	0.81–3.24			0.84	0.51–1.41		
Female Gender	0.26	0.05–1.27			1.42	0.60–3.37		
CD4 baseline	1.09	0.61–1.92			1.38	0.92–2.08		
Log viral load baseline	2.34	0.76–7.15			0.77	0.42–1.42		
HbA1c	1.83	0.96–3.49			1.43	0.94–2.16		
Sputum TTP	1.38	0.53–3.63			0.78	0.43–1.49		
Smoking (current)	2.60	0.53–12.94			1.08	0.32–3.67		
Smoking (ever)	3.90	0.94–16.15			1.37	0.45–4.13		

Age categorized into < 30, 30–39, 40–49, ≥ 50; Baseline CD4 categorized into < 50, 50–99, 100–199, ≥ 200; HbA1c categorized into < 5.7, 5.7–5.9, 6.0–6.2, ≥ 6.3; CAT score categorized into < 5, 5–9, 10–14, ≥ 15; CT involvement categorized into < 10, 10–14, ≥ 15; Sputum TTP categorized into < 10, 10–14, 15–19, ≥ 20

*PFT* pulmonary function test, *OR* odds ratio, *95% CI* 95% confidence interval, *HbA1c* hemoglobin A1c, *CAT* COPD assessment test, *TTP* time to positivity, *CT* computed tomography

^a^Adjusted for age, gender, baseline CD4, smoking. Statistically significant associations are indicated in bold

### Lung function is dynamic over the course of tuberculosis treatment

There were 93 participants who completed tuberculosis treatment and underwent spirometry testing at six months, 85 (91%) of whom were able to complete satisfactory testing. At that time, there were 51 (60%) participants with normal spirometry, 14 (16%) with obstruction, 17 (20%) with restriction, and three (4%) with a mixed pattern. Six months later, 12 months after diagnosis, spirometry was performed for 95 participants, 92 (97%) of whom completed satisfactory testing; 62 (67%) had normal pulmonary function, 10 (11%) had obstruction, 14 (15%) had restriction, and 6 (7%) had a mixed pattern. Among the 10 participants with an obstructive pattern, the median FEV_1_% predicted was 76.5 (IQR 64–85) and only 3 (30%) participants had an FEV_1_ less than 70% of predicted.

**Fig. 2:**
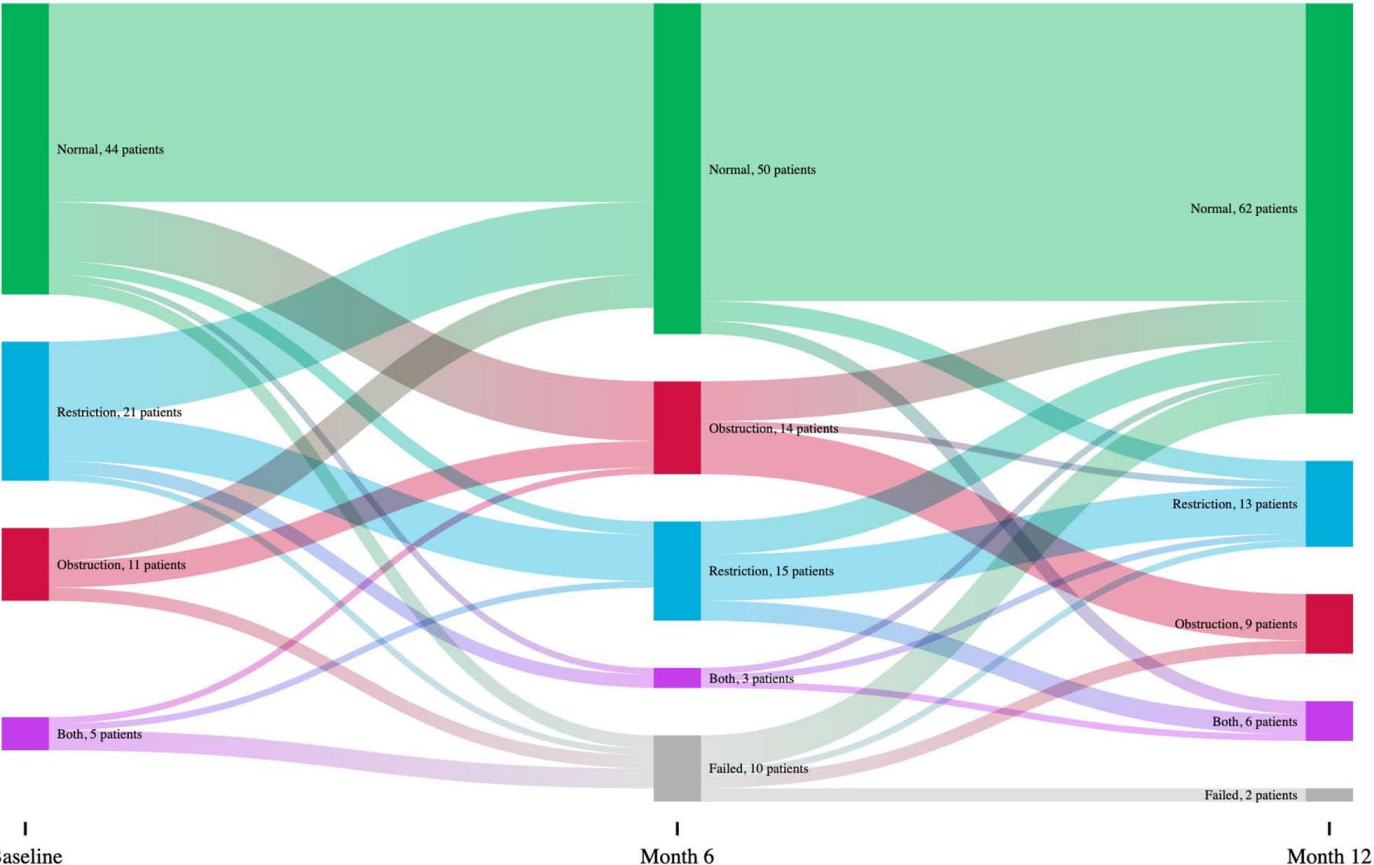
Changes in pulmonary diagnoses at baseline, 6 months and 12 months. For 44 participants with a normal baseline pattern, 35 (80%) remained normal at 12 months, while 6 (14%) developed incident obstruction, 1 (2%) developed restriction, and 2 (5%) developed a mixed pattern. For 10 participants with obstruction at baseline, 7 (70%) had normalized at 12 months, while 3 (30%) had persistent obstruction. For 22 participants with restriction at baseline, 12 (55%) had normalized at 12 months, while 8 (36%) had persistent restriction, 1 (5%) had developed obstruction in addition to restriction (i.e., a mixed pattern), and 1 (5%) had obstruction alone. For 5 participants who had a mixed pattern both at baseline, 2 (40%) had normalized at 12 months, 2 (40%) had persistent restriction, and 1 (20%) had a persistent mixed pattern

Among 81 participants with spirometry at both baseline and 12 months, we examined the changes in spirometric patterns over time (Fig. 2). We found that baseline spirometric diagnosis had poor agreement with 12-month diagnosis (κ = 0.26, *p* value < 0.001) and 6-month diagnosis had only moderate agreement with 12-month diagnosis (κ = 0.50, *p* value < 0.001). For 44 participants with a normal baseline pattern, 35 (80%) remained normal at 12 months. Among 10 participants with obstruction at baseline, 7 (70%) had normalized at 12 months, while among 22 participants with restriction at baseline, only 12 (55%) had normalized at 12 months.

Over the course of the study, six participants met criteria for TB-IRIS [27]; one participant had worsening dyspnea in the setting of a new pleural effusion and five participants had worsening lymphadenopathy in either the cervical or abdominal regions. There was no apparent association between lung function and the development of clinical IRIS in this limited number of participants. No other opportunistic infections were identified using routine clinical screening.

### Pulmonary symptoms are uncommon after tuberculosis treatment

Only 12 (13%) participants reported symptoms at 12 months, including seven (58%) participants with normal spirometry at that time, three (25%) with restriction, and two (17%) with a mixed pattern. Although participants with restriction reported more symptoms than those with obstruction or normal spirometry, this difference was not statistically significant (*p* value 0.10; Additional file 1: Table S4). There was also no significant difference in 6 min walk test distance for those with and without normal pulmonary function at 12 months.

### Pulmonary restriction at baseline is associated with long-term pulmonary impairment

In bivariate logistic regression analyses, having either a restrictive or mixed pattern of pulmonary impairment at baseline was associated with having abnormal pulmonary function at 12 months (OR for baseline restriction 3.24, 95% 1.06–9.87; OR for baseline mixed 5.83, 95% CI 0.84–40.32) (Table 5). After adjustment for age, gender, change in CD4 count from baseline to week 4, and time from TB treatment to ART initiation, baseline restriction and mixed pattern remained significantly associated with impaired pulmonary function at 12 months (aOR for baseline restriction 5.44, 95% CI 1.16–25.45; aOR for baseline mixed 9.00, 95% CI 1.05–76.87).

**Table 5:** Unadjusted and adjusted odds ratios for the association between baseline pulmonary impairment and any pulmonary impairment at 12 months

	Unadjusted OR	95% CI	Adjusted OR^a^	95% CI
Baseline normal lung function	Ref		Ref	
Baseline obstruction	1.67	0.36–7.76	1.95	0.28–13.78
Baseline restriction	**3.24**	**1.06–9.87**	**5.44**	**1.16–25.45**
Baseline mixed	**5.83**	**0.84–40.32**	**9.00**	**1.05–76.87**
Age	1.31	0.76–2.23		
Female Gender	1.22	0.51–2.91		
CD4 baseline	1.10	0.74–1.64		
CD4 change from baseline to week 4	0.81	0.53–1.23		
Log viral load baseline	0.79	0.42–1.47		
Log viral load change from baseline to week 4	1.09	0.70–1.71		
Baseline HbA1c	1.13	0.76–1.69		
Baseline CAT score	1.24	0.82–1.87		
Final CAT score	1.51	0.44–5.22		
Sputum TTP	0.94	0.51–1.72		
Smoking (current)	0.79	0.24–2.57		
Smoking (ever)	0.95	0.31–2.91		
Time from TB treatment to ART	**1.02**	**1.00–1.04**		

Age: Categorized into < 30, 30–39, 40–49, > 50; Baseline CD4 categorized into < 50, 50–99, 100–199, > 200; CAT score < 5, 5–9, 10–15, > 15; HbA1c categorized into < 5.7, 5.7–5.9, 6.0–6.2, ≥ 6.3; CT involvement < 10, 10–15, > 15; TTP < 10, 10–15, 15–20, > 20

*PFT* pulmonary function test, *OR* odds ratio, *95% CI* 95% confidence interval, HbA1c hemoglobin A1c, *CAT* COPD assessment test, *TTP* time to positivity, *TB tx* tuberculosis treatment, *ART* antiretroviral therapy, *CT* computed tomography

^a^Adjusted for age, gender, change in CD4 count from baseline to week 4, time from TB treatment to ART initiation. Statistically significant associations are indicated in bold

In a second set of models that assessed the odds of having either restriction or obstruction at 12 months, impaired pulmonary function at baseline was strongly associated with restriction, but not obstruction at 12 months (OR for obstruction 1.11, 95% CI 0.28–4.40; OR for restriction 16.67, 95% CI 1.99–139.64) (Additional file 1: Table S6). Multivariate models for modeling restriction or obstruction at 12 months were unable to converge on a maximum likelihood estimate. Baseline symptoms were not associated with impaired pulmonary function at 12 months in any of the models.

## Discussion

In this study we found that half of individuals with tuberculosis and HIV coinfection had impaired pulmonary function at the time of tuberculosis diagnosis and one-third had impaired pulmonary function 12 months later, 6 months after finishing their tuberculosis treatment. At every time point, a restrictive pattern was more common than obstruction. Impaired pulmonary function at baseline, specifically in a restrictive pattern, was significantly associated with greater pulmonary symptoms. Furthermore, pulmonary restriction at baseline was associated with persistently impaired pulmonary function at 12 months, when lung impairment may be permanent.

Our finding that restriction was the most commonly identified spirometric abnormality is similar to what has been reported from a cohort in India, where 52% of individuals had restriction and 24% had obstruction [10]. In contrast to India where obstruction was more symptomatic than restriction, we found that individuals with restriction were more symptomatic than those with obstruction alone. However, HIV prevalence in the cohort from India was only 4% and so it is possible that differences in the HIV coinfection rates may explain some of the differences in our findings. Nevertheless, greater symptoms among the patients with restriction may relate to the distribution and amount of lung involvement at presentation, as those with restriction also had greater radiologic involvement than those with normal spirometry or obstruction. While bronchiectasis was associated with both obstruction and restriction, a number of additional radiologic abnormalities, including bronchiolitis, consolidation, and mosaic perfusion, were significantly more common among those with restriction. In the context of tuberculosis, these findings suggest that airway disease is common, but only a subset of individuals with sufficient parenchymal involvement may be at risk for developing a restrictive ventilatory defect.

While restriction has been reported among tuberculosis survivors without HIV, people with HIV may have a greater propensity to develop parenchymal involvement and, by extension, restrictive lung disease. Higher rates of emphysema and pulmonary fibrosis in people with HIV have been ascribed to the deleterious effects of virally mediated chronic inflammation and oxidative stress [28, 29]. Hence, it is possible that those same perturbations increase the risk of post-tuberculosis pulmonary fibrosis. People with HIV also have a relative CD8 cell alveolitis, which may further skew the pulmonary immune response towards fibrosis [30–32].

Impaired lung function at baseline, specifically a restrictive or mixed pattern, was a strong predictor for impaired lung function at 12 months. However, 20% of those with normal spirometry at baseline went on to develop incident pulmonary impairment at 12 months. These data suggest that there may be two phases of lung damage in patients with HIV and tuberculosis—a first phase prior to diagnosis and a second phase after treatment initiation, that may represent a pulmonary-specific manifestation of immune reconstitution inflammatory syndrome (IRIS) [17]. Interventions to prevent lung injury may differ according to these phases. Lung damage that occurs prior to diagnosis will best be prevented by continuing to emphasize early diagnosis and active case finding, whereas lung damage that occurs after treatment initiation may be amenable to intervention with anti-inflammatory agents or other host-directed therapies. In light of the predominance of restrictive lung disease in this cohort, anti-fibrotic agents, which are increasingly utilized for interstitial lung diseases, represent a promising avenue for preventing pulmonary impairment after tuberculosis [33, 34].

Given that respiratory symptoms are often one of the only indications that someone may have decreased lung function, we were surprised to find that few participants reported symptoms at 12 months, irrespective of whether they had impaired pulmonary function. It is possible that these participants may have minimized their pulmonary symptoms, particularly after having been treated for tuberculosis and initiating ART. Another possibility is that these individuals, who were generally quite young, may be able to compensate for their impaired lung function but will become more symptomatic as they age. Yet another consideration is that the COPD Assessment Test is not a sensitive tool for identifying pulmonary symptoms related to tuberculosis. Future studies could assess other measures of exercise capacity, such as the incremental shuttle walk test, among tuberculosis survivors [35].

There are several limitations to this study. Firstly, spirometry is not accurate for diagnosing restrictive lung physiology, with a positive predictive value of 58–64% when compared to the standard of plethysmography [36, 37]. However, equipment for plethysmography is relatively expensive and thus is difficult to obtain in resource-limited settings. Lung volume measurements with nitrogen washout or single-breath helium dilution present alternatives that are more cost-effective but may underestimate lung volumes and still require additional equipment [38]. Spirometry is also effort dependent, and so improvements in testing over the course of treatment for tuberculosis and HIV could reflect improvements in overall health, and not simply improvements in lung function. A second limitation is the use of low-dose, thick-section CT scans to assess for radiologic involvement. While these images do not provide the same resolution of pulmonary anatomy as high-resolution diagnostic scans, they do offer substantially greater detail than chest radiographs. Third, we cannot be certain that the observed pulmonary dysfunction was solely due to tuberculosis, as this is an immunocompromised cohort at risk for concurrent infections and because pre-tuberculosis spirometry data were not available. Finally, while we were able to follow participants for up to 6 months after completion of tuberculosis treatment, given the flux in spirometric diagnoses between 6 and 12 months, there could be ongoing pulmonary remodeling even after 12 months.

## Conclusions

In light of growing global calls to better understand pulmonary impairment after tuberculosis, our findings from this prospective study make an important contribution to the literature [39]. We found that pulmonary impairment is common in people with HIV coinfection who are successfully treated for pulmonary tuberculosis, even up to 6 months after treatment completion. Further, restriction is not only the most common pattern on spirometry, but is also the pattern most commonly associated with respiratory symptoms and most predictive of long-term pulmonary impairment. While the participants enrolled in this study had advanced HIV disease with a median CD4 count of 110 cells/μL, they also represent a young cohort with minimal smoking history who would otherwise be expected to have little underlying pulmonary disease. Our finding that one-third of these individuals have impaired lung function 6 months after successful completion of tuberculosis treatment is highly concerning, as these impairments will likely impact their quality of life for decades to come. There is an urgent need to better understand pulmonary impairment after tuberculosis—from risk factors and predictors of lung injury to biological pathways that may be amenable to pharmacologic intervention—to lessen the burden of chronic lung impairment in this vulnerable population.

## Supplementary Information

**Additional file 1.** Additional file contains supplementary methods regarding the spirometry testing and additional Tables as referenced in the manuscript.

## Data Availability

The datasets used and/or analysed during the current study are available from the corresponding author on reasonable request.

## Abbreviations

6MWT: 6 Min walk test
aOR: Adjusted odds ratio
ART: Antiretroviral therapy
BMI: Body mass index
CAT: COPD assessment test
CI: Confidence interval
COPD: Chronic obstructive pulmonary disease
CT: Computed tomography
FDG: Fluorodeoxyglucose
FEV_1_: Forced expiratory volume in 1 s
FVC: Forced vital capacity
GOLD: Global initiative for chronic obstructive lung disease
HbA1c: Hemogloblin A1c
IQR: Interquartile range
IRIS: Immune reconstitution inflammatory syndrome
LIFT-IRIS: Lung function after TB-IRIS
OR: Odds ratio
PET: Positron emission tomography
PFT: Pulmonary function test
TTP: Time to positivity
