# Supplementary material for: Pulmonary restriction predicts long-term pulmonary impairment in people with HIV and tuberculosis
Source: BMC Pulm Med. 2021 Jan 7;21:19. doi: 10.1186/s12890-020-01368-4 (PMC7791797; doi:10.1186/s12890-020-01368-4)

**Pulmonary restriction predicts long-term pulmonary impairment in people with HIV and tuberculosis**

Sara C. Auld MD, MS,^1^ Hardy Kornfeld MD,^2^ Pholo Maenetje PhD,^3^ Mandla Mlotshwa PhD,^3^ William Chase BS,^4^ Mboyo di-Tamba Vangu MD, MS, PhD,^5^ Drew A. Torigian MD,^4^ Robert S. Wallis MD,^3^ Gavin Churchyard PhD,^3,6^ Gregory P. Bisson MD, MS^4,7^

**SUPPLEMENTARY MATERIALS**

**Supplementary Methods:**

All spirometry testing was done without the administration of bronchodilator therapy. Single-use ‘barriette’ and ‘spirette’ pieces were used to provide hygienic separation of patients from the analyzer and address tuberculosis transmission risk. Other spirometry system components were decontaminated between tests. Spirometry maneuvers were included in the analysis if acceptable by ATS standards and without gross anomalies on the flow-volume loops (e.g., glottic closure, large cough). Both absolute volumes (liters) and percent predicted values (adjusted for age, race, height) were collected for the forced expiratory volume in 1 second (FEV1) and forced vital capacity (FVC). The number of acceptable maneuvers performed at each spirometry session are documented in Table S1.

In addition to the fixed threshold classification of obstruction, restriction, and mixed patterns, we also classified participants as having obstruction or restriction on the basis of an FEV_1_/FVC ratio or FVC less than the lower limit of normal (LLN), which corresponds to values below the 5^th^ percentile of expected values.^1^ We found excellent agreement between diagnoses according to the fixed-ratio and LLN definitions (κ = 0.86, p-value < 0.0001; Table S2).

PET/CT scans were conducted at baseline using an integrated 40-detector scanner (Biograph 40 True Point PET/CT, Siemens Medical, IL, USA) with 1.5mm slice thickness, 120 kilovoltage, and 26-30 milliampere-second during quiet breathing. For the present study, CT images from these scans were evaluated using a modified version of a score developed for non-tuberculous mycobacterial diseases correlated with reduced lung volumes and flow rates (Table S3).^2^ Scans were scored for bronchiectasis, i.e., enlargement of the bronchial lumen with or without bronchial wall thickening or mucus plugging, bronchiolitis, cavity, nodules, consolidation, bullae, emphysema, mosaic perfusion, lobar volume decrease, and pleural effusion by a board-certified chest radiologist (DAT) who was blinded to all other clinical data.

**References:**

1. Pellegrino R, Viegi G, Brusasco V, et al. Interpretative strategies for lung function tests. *The European respiratory journal* 2005; **26**(5): 948-68.

2. Song JW, Koh WJ, Lee KS, et al. High-resolution CT findings of Mycobacterium avium-intracellulare complex pulmonary disease: correlation with pulmonary function test results. *AJR American journal of roentgenology* 2008; **191**(4): W160.

Table S1. Number of acceptable spirometry maneuvers per visit.

|  | Total with spirometry (n) | 1 spirometry maneuver (n, %) | 2 spirometry maneuvers (n, %) | 3 spirometry maneuvers (n, %) |
| --- | --- | --- | --- | --- |
| Baseline visit | 112 | 18 (16) | 25 (22) | 68 (61) |
| 6 month visit | 85 | 4 (5) | 15 (18) | 66 (78) |
| 12 month visit | 92 | 8 (9) | 12 (13) | 72 (78) |

Table S2. Comparison of diagnoses using fixed ratio vs. lower limit of normal (LLN) thresholds. Kappa = 0.86 (p-value < 0.001).

|  |  | LLN definitions |  |  |  |  |
| --- | --- | --- | --- | --- | --- | --- |
|  |  | Normal | Obstruction | Restriction | Both | Total |
| Fixed ratio definitions | Normal | 251 | 8 | 3 | 0 | 262 |
|  | Obstruction | 13 | 46 | 0 | 2 | 61 |
|  | Restriction | 7 | 0 | 103 | 1 | 111 |
|  | Both | 0 | 2 | 3 | 27 | 32 |
|  | Total | 271 | 56 | 109 | 30 | 466 |

Table S2. CT scoring system (Adapted from Song et al, American Journal of Roentgenology 2008)

|  | Score |  |  |  |
| --- | --- | --- | --- | --- |
| CT Finding | 0 | 1 | 2 | 3 |
| Bronchiectasis (12 points) |  |  |  |  |
| Severity | Absent | Mild (bronchus diameter >  adjacent vessel diameter) | Moderate (bronchus diameter = 2–3× vessel diameter) | Severe (bronchus diameter  > 3× vessel diameter) |
| Extent | Absent | 1–5 segments | 6–9 segments | > 9 segments |
| Bronchial wall thickening | Absent | Mild (wall thickness =  adjacent vessel diameter) | Moderate (vessel diameter  < wall thickness < 2× vessel  diameter) | Severe (wall thickness ≥ 2×  vessel diameter) |
| Mucus plugging | Absent | 1–5 segments | 6–9 segments | > 9 segments |
| Bronchiolitis (6 points) |  |  |  |  |
| Severity | Absent | Mild (identifiable;  peripheral lung < 2 cm  from pleura) | Moderate (definite; involvement > 2 cm from pleura) | Severe (extensive;  extending to central lung) |
| Extent | Absent | 1–5 segments | 6–9 segments | > 9 segments |
| Cavity (6 points) |  |  |  |  |
| Severity | Absent | Mild (diameter < 3 cm) | Moderate (3 cm < diameter  < 5 cm) | Severe (diameter ≥ 5 cm) |
| Extent | Absent | 1–3 in number | 4–5 in number | > 5 in number |
| Nodules (10-30mm in diameter) (3 points) | Absent | 1–5 segments | 6–9 segments | > 9 segments |
| Consolidation, lobular, segmental, or peribronchial (3 points) | Absent | < 3 segments | 3-5 segments | > 5 segments |
| Bullae (3 points) | Absent | Unilateral (< 4 in number) | Bilateral (< 4 in number) | Bilateral (≥ 4 in number) |
| Emphysema (3 points) | Absent | 1–5 segments | > 5 segments | NA |
| Mosaic perfusion (3 points) | Absent | 1–5 segments | > 5 segments | NA |
| Lobar volume decrease (3 points) | Absent | 1 lobe | 2 lobes | ≥ 3 lobes |
| Pleural effusion (2 points) | Absent | Unilateral | Bilateral | NA |

Table S3. Pulmonary symptoms according to patterns of lung function at 12 months. (Median and IQR)

|  | **Normal  (n = 62)** | **Obstruction (n = 10)** | **Restriction (n = 14)** | **Both (n = 6)** |
| --- | --- | --- | --- | --- |
| Total CAT score | 0 (0-0) | 0 (0-0) | 0 (0-0) | 0 (0-1) |
| CAT 0  n (%) | 55 (89) | 10 (100) | 11 (79) | 4 (67) |
| CAT 1-5 | 7 (11) | 0 (0) | 2 (14) | 2 (33) |
| CAT 6-10 | 0 (0) | 0 (0) | 1 (7) | 0 (0) |
| 6MWT distance | 434  (391-480) | 412  (386-455) | 405  386-445) | 481  (448-516) |

Table S4. Baseline CT abnormalities and patterns of lung function at 12 months. (Median and IQR)

|  | Normal (17) | Obstruction (3) | Restriction (6) | Both  (3) | P* | P** | P*** | P**** | P***** |
| --- | --- | --- | --- | --- | --- | --- | --- | --- | --- |
| Total CT score | 11 (9-17) | 15 (3-25) | 18 (11-28) | 16 (10-21) | 0.56 | 0.18 | 0.71 | 0.21 | 0.15 |
| Bronchiectasis | 2 (2-5) | 8 (1-8) | 7 (4-11) | 5 (1-8) | 0.24 | 0.07 | 0.48 | **0.04** | 0.07 |
| Bronchiolitis | 4 (2-6) | 5 (2-6) | 6 (5-6) | 4 (3-6) | 0.69 | 0.34 | 0.79 | 0.23 | 0.30 |
| Cavities | 0 (0-2) | 0 (0-2) | 2 (0-4) | 0 (0-2) | 0.68 | 0.69 | 0.76 | 0.31 | 0.52 |
| Nodules | 1 (1-1) | 1 (0-3) | 0 (0-1) | 1 (1-1) | 0.20 | 0.42 | 0.71 | 0.06 | 0.23 |
| Consolidation | 1 (1-2) | 1 (0-2) | 2 (1-3) | 1 (0-2) | 0.82 | 0.61 | 0.78 | 0.42 | 0.45 |
| Bullae | 0 (0-0) | 0 (0-2) | 0 (0-0) | 0 (0-2) | 0.51 | 0.75 | 0.63 | 0.20 | 0.50 |
| Emphysema | 0 (0-0) | 0 (0-1) | 0 (0-0) | 0 (0-1) | 0.93 | 0.93 | 0.72 | 0.73 | 0.94 |
| Mosaic perfusion | 2 (1-2) | 0 (0-1) | 2 (1-2) | 2 (1-2) | 0.07 | 0.20 | **0.01** | 0.62 | 0.75 |
| Lobar volume decrease | 0 (0-0) | 0 (0-0) | 1 (0-2) | 0 (0-3) | 0.07 | 0.10 | 0.44 | **0.02** | **0.03** |
| Pleural effusion | 0 (0-0) | 0 (0-0) | 1 (0-1) | 0 (0-1) | **0.02** | **0.01** | 1 | **0.002** | **0.003** |

*p-value for all groups (Kruskal Wallis test); **p-value for normal vs. abnormal (O+R+B); ***p-value for normal vs. obstruction; ****p-value for normal vs. restriction; *****p-value for normal vs. restriction+both

Table S5. Logistic regression for obstruction or restriction at baseline.

|  | Baseline obstruction | | | | | | Baseline restriction | | | | | |
| --- | --- | --- | --- | --- | --- | --- | --- | --- | --- | --- | --- | --- |
|  | Univariate | 95% CI | p-value | Multivariate | 95% CI | p-value | Univariate | 95% CI | p-value | Multivariate | 95% CI | p-value |
| Age | 1.62 | 0.81-3.24 | 0.17 | 1.53 | 0.70-3.41 | 0.30 | 0.85 | 0.51-1.42 | 0.54 |  |  |  |
| Female Gender | 0.26 | 0.05-1.27 | 0.10 | 0.75 | 0.11-5.26 | 0.77 | 1.44 | 0.60-3.44 | 0.42 |  |  |  |
| CD4 baseline | 1.09 | 0.61-1.92 | 0.78 |  |  |  | 1.33 | 0.88-2.01 | 0.17 | 1.29 | 0.83-2.00 | 0.26 |
| Log VL baseline | 2.34 | 0.76-7.15 | 0.14 | 1.86 | 0.52-6.67 | 0.34 | 0.78 | 0.42-1.44 | 0.42 |  |  |  |
| HbA1c | 1.83 | 0.96-3.49 | 0.07 | 1.48 | 0.70-3.14 | 0.31 | 1.43 | 0.94-2.16 | 0.10 | 1.49 | 0.93-2.38 | 0.10 |
| CAT score | 0.99 | 0.53-1.84 | 0.97 |  |  |  | **1.79** | **1.18-2.72** | **0.01** | **1.66** | **1.08-2.54** | **0.02** |
| Sputum TTP | 1.38 | 0.53-3.63 | 0.51 |  |  |  | 0.79 | 0.41-1.52 | 0.49 |  |  |  |
| Smoking (current) | 2.60 | 0.52-12.94 | 0.24 | 1.65 | 0.27-9.94 | 0.59 | 1.03 | 0.30-3.49 | 0.96 |  |  |  |
| Smoking (ever) | 3.90 | 0.94-16.15 | 0.06 | 3.10 | 0.62-15.35 | 0.17 | 1.30 | 0.43-3.92 | 0.65 |  |  |  |
|  |  |  |  |  |  |  |  |  |  |  |  |  |
|  |  |  |  |  |  |  |  |  |  |  |  |  |
| Baseline CT involvement | 2.98 | 0.76-11.67 | 0.12 |  |  |  |  |  |  |  |  |  |

Table S6. Unadjusted odds ratios for the association between baseline pulmonary impairment and pulmonary obstruction or restriction as compared to normal lung function at 12 months.

|  | Obstruction at 12 months | | Restriction at 12 months | |
| --- | --- | --- | --- | --- |
|  | Unadjusted OR | 95% CI | Unadjusted OR | 95% CI |
| Impaired baseline lung function | 1.11 | 0.28-4.40 | **16.67** | **1.99-139.64** |
| Age | 2.33 | 0.99-5.45 | 0.74 | 0.35-1.57 |
| Female Gender | 0.37 | 0.07-1.89 | **4.07** | **1.16-14.23** |
| CD4 baseline | 0.79 | 0.42-1.49 | 1.59 | 0.89-2.84 |
| CD4 change from baseline to week 4 | 0.91 | 0.48-1.71 | 0.76 | 0.45-1.29 |
| Log viral load baseline | 1.26 | 0.44-3.62 | 0.57 | 0.25-1.28 |
| Log viral load change from baseline to week 4 | 0.72 | 0.34-1.52 | 1.91 | 0.94-3.87 |
| Baseline HbA1c | 1.07 | 0.58-1.99 | 1.16 | 0.69-1.98 |
| Baseline CAT score | 0.80 | 0.39-1.64 | 1.37 | 0.82-2.28 |
| Sputum TTP | 1.13 | 0.39-3.29 | 0.72 | 0.33-1.54 |
| Smoking (current) | 2.53 | 0.59-10.98 | -- | -- |
| Smoking (ever) | 0.63 | 0.07-5.97 | 0.79 | 0.19-3.28 |
| Time from TB treatment to ART | **1.06** | **1.01-1.11** | 1.00 | 0.97-1.04 |

*Age: Categorized into <30, 30-39, 40-49, >50; Baseline CD4 categorized into < 50, 50-99, 100-199, > 200; CAT score <5, 5-9, 10-15, >15; HbA1c categorized into < 5.7, 5.7-5.9, 6.0-6.2, ≥ 6.3; CT involvement <10, 10-15, >15; TTP <10, 10-15, 15-20, >20*

Figure S1. Total CAT symptom score plotted against (a) FEV_1_ % predicted and (b) FVC % predicted at baseline.


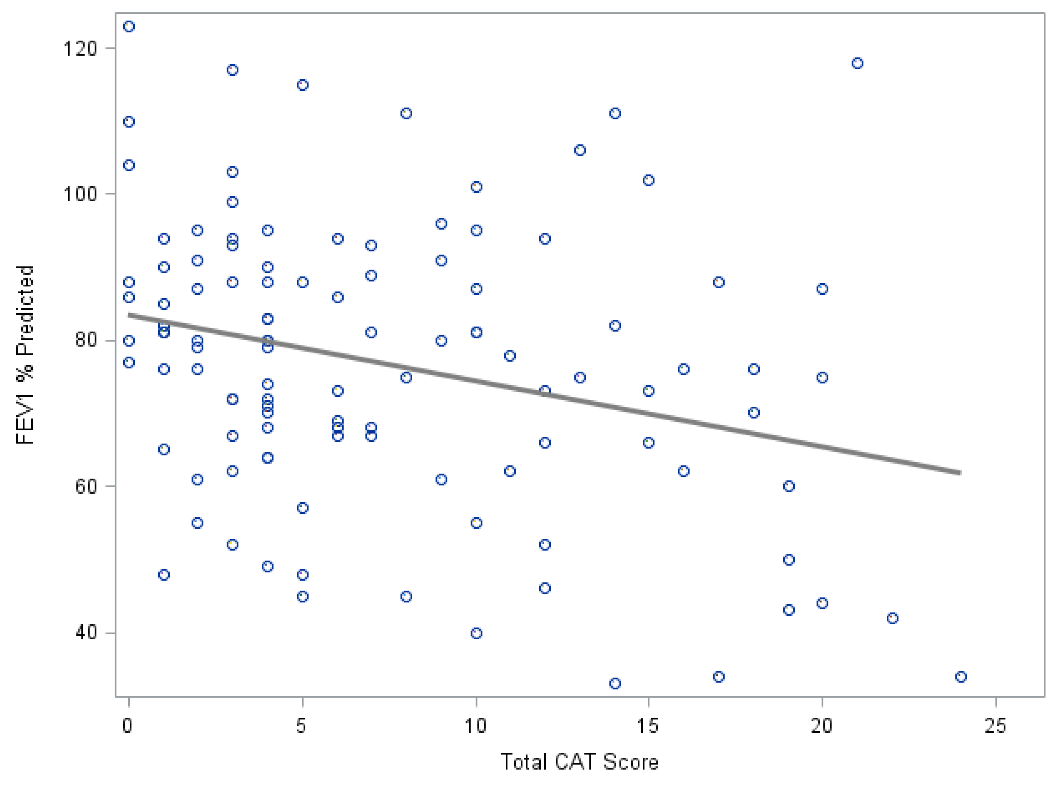

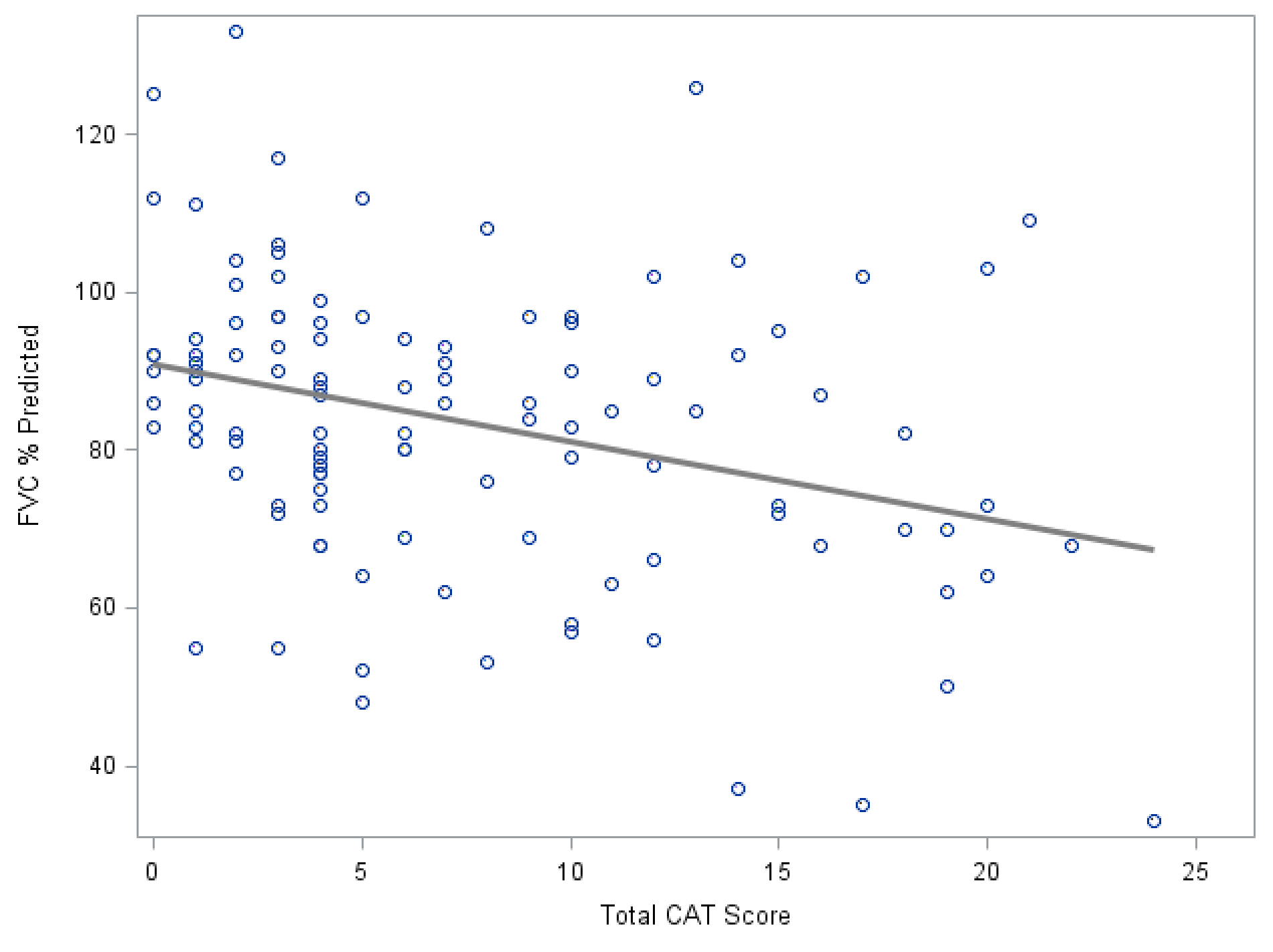


Figure S2. Total CT score plotted against (a) FEV_1_ % predicted and (b) FVC % predicted at baseline.


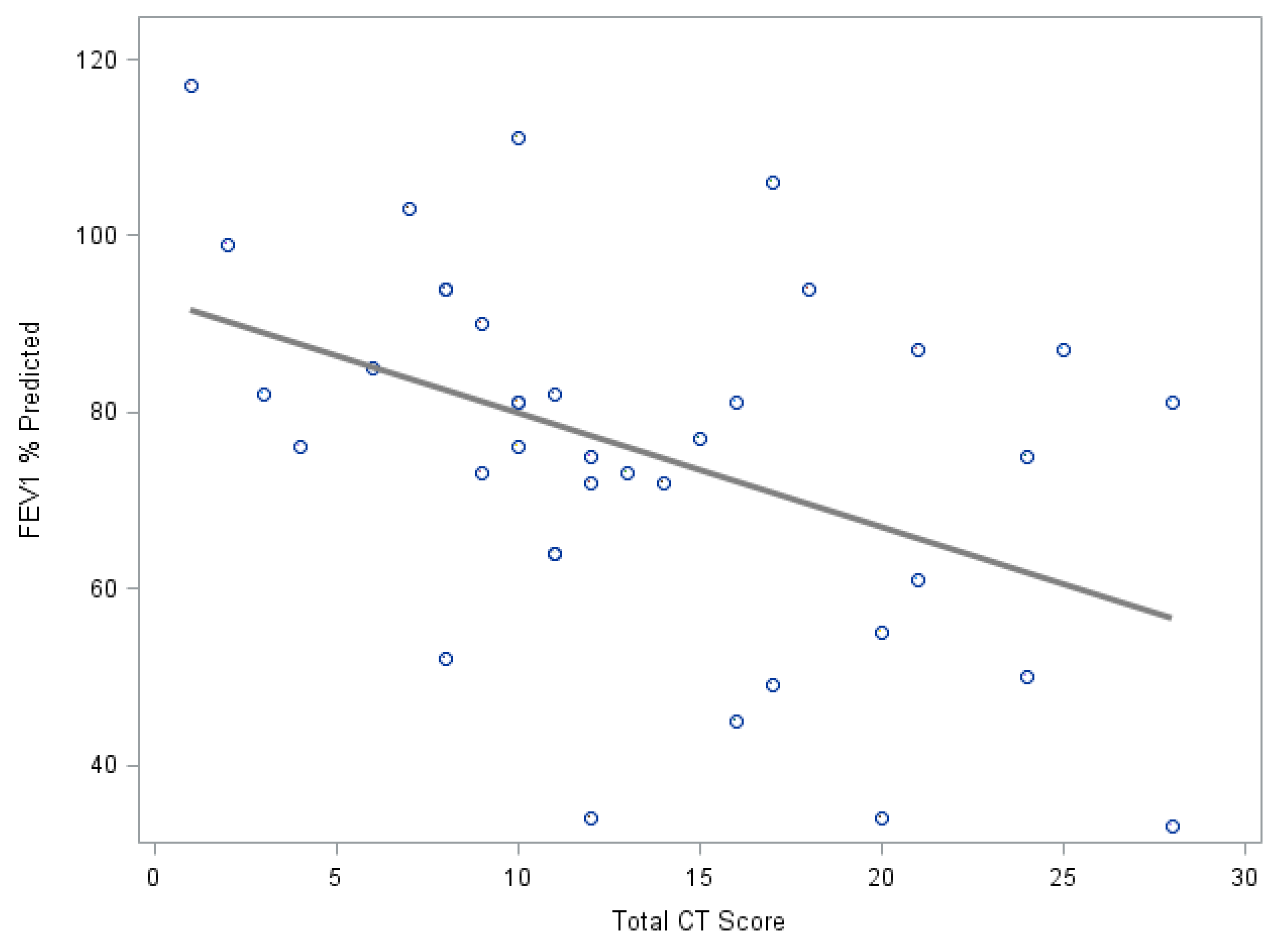

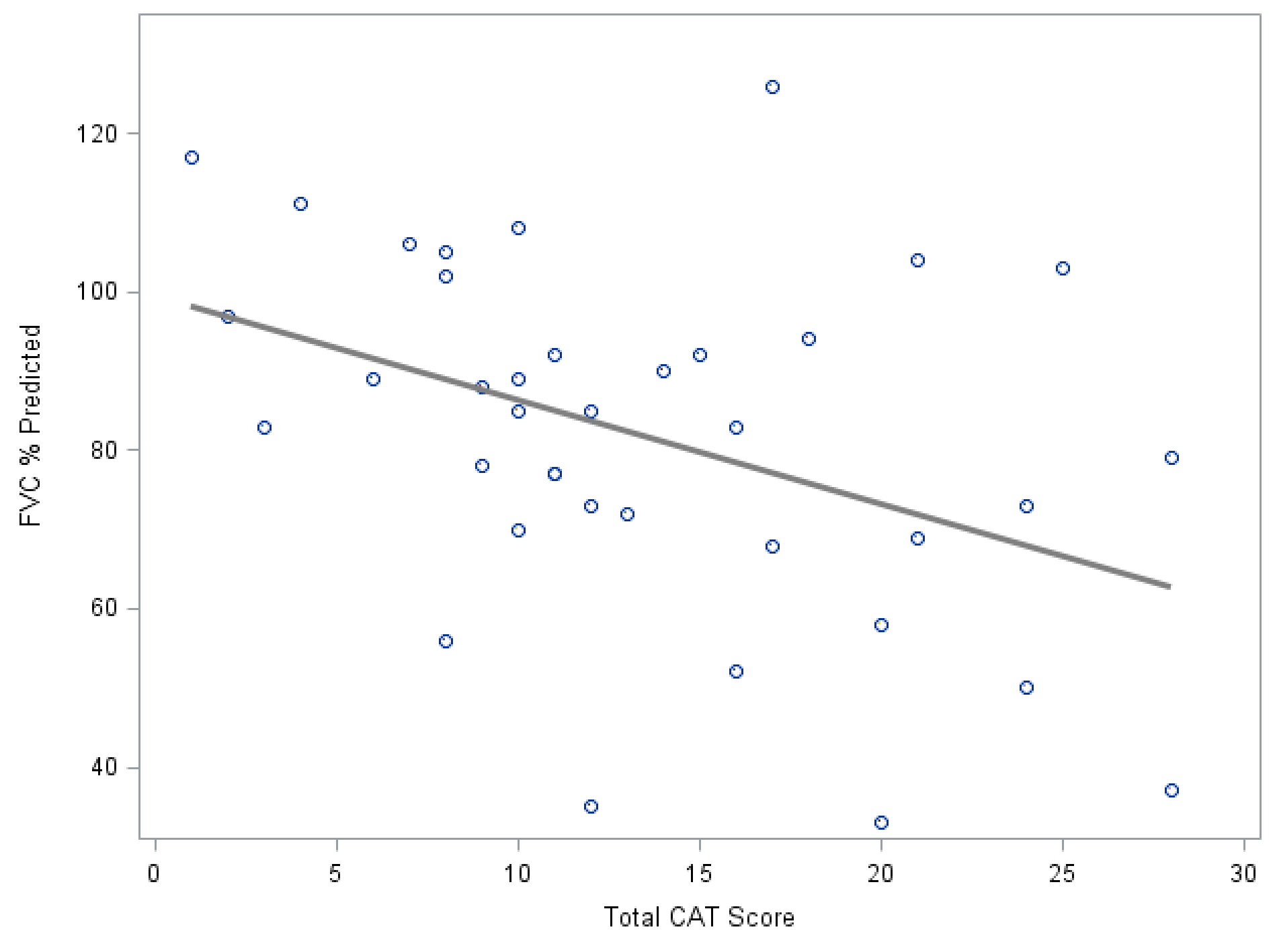

Supplement: Supplementary file 1 — Additional file 1. Additional file contains supplementary methods regarding the spirometry testing and additional Tables as referenced in the manuscript. [file 12890_2020_1368_MOESM1_ESM.docx]
